# Complete Genome Sequences of Three Multidrug-Resistant Clinical Isolates of *Streptococcus pneumoniae* Serotype 19A with Different Susceptibilities to the Myxobacterial Metabolite Carolacton

**DOI:** 10.1128/genomeA.01641-16

**Published:** 2017-02-16

**Authors:** Jannik Donner, Boyke Bunk, Isabel Schober, Cathrin Spröer, Simone Bergmann, Michael Jarek, Jörg Overmann, Irene Wagner-Döbler

**Affiliations:** a Department of Medical Microbiology, Research Group Microbial Communication, Helmholtz Centre for Infection Research, Braunschweig, Germany; b Leibniz Institute DSMZ-German Collection of Microorganisms and Cell Cultures, Braunschweig, Germany and German Centre for Infection Research (DZIF), Partner Site Hannover–Braunschweig, Braunschweig, Germany; c Technische Universität Braunschweig, Institute of Microbiology, Braunschweig, Germany; d Genome Analytics, Helmholtz Centre for Infection Research, Braunschweig, Germany

## Abstract

The full-genome sequences of three drug- and multidrug-resistant *Streptococcus pneumoniae* clinical isolates of serotype 19A were determined by PacBio single-molecule real-time sequencing, in combination with Illumina MiSeq sequencing. A comparison to the genomes of other pneumococci indicates a high nucleotide sequence identity to strains Hungary19A-6 and TCH8431/19A.

## GENOME ANNOUNCEMENT

*Streptococcus pneumoniae* (pneumococcus), a frequent colonizer of the human naso-oropharynx, causes severe invasive and noninvasive infections in susceptible patients (1, 2). Clinical pneumococcal isolates were obtained from patients suffering from septic pneumonia (SP49) or pleuritis (SP61) and from the auditory canal of an asymptomatic patient (SP64) (University Hospital Aachen, Germany). They were serotyped and tested for antimicrobial susceptibility at the German Reference Center for Streptococci (Aachen). The strains differed strikingly in their susceptibility to the macrolide ketocarbonic acid carolacton: while SP49 was highly susceptible, growth of SP61 and SP64 was only slightly inhibited (3).

Bacterial DNA was isolated using the NucleoSpin tissue kit (Macherey-Nagel) and processed for PacBio single-molecule real-time (SMRT) sequencing and Illumina MiSeq paired-end sequencing (2 × 250 bp). The read count obtained during SMRT sequencing varied between 48,710 and 119,441 reads/sample, resulting in a 114- to 180-fold coverage of the genomes. *De novo* genome assemblies were constructed with PacBio’s SMRT Portal version 2.3.0 using the Hierarchical Genome Assembly Process (HGAP3) (4). Insertion/Deletion (InDel) errors were corrected by mapping of Illumina reads onto finished genomes using Burrows–Wheeler alignment (5) with subsequent variant and consensus calling using VarScan (6); automated sequence annotation was performed by Prokka version 1.8 (7).

The genome sequences of SP49, SP61, and SP64 were 2,206,644 bp, 2,071,812 bp, and 2,073,113 bp in length and contained 2,183, 2,025, and 2,024 coding sequences, respectively. The G+C content was consistently 39.9%.

The three genomes were compared to all 29 complete pneumococcal genomes publicly available at the NCBI. SP49 showed the highest *in silico* DNA-DNA hybridization (*is*DDH) values (>86%) to* S. pneumoniae* Hungary19A-6 (NC_010380.1), while SP61 and SP64 were most similar (>99% *is*DDH) to *S. pneumoniae* TCH8431/19A (NC_014251.1), as calculated by the Genome-to-Genome Distance Calculator (GGDC) version 2.1 (8).

*S. pneumoniae* Hungary19A-6 and TCH8431/19A are virulent strains of serotype 19A and are often associated with invasive pneumococcal disease and antibiotic resistance (9, 10).

According to ARG-ANNOT (11), the genomes of all pneumococcal isolates carry resistance loci that coincide with their recorded antibiotic resistance phenotypes (3). They encode mutations in genes of penicillin-binding proteins, causing amino acid substitutions known to mediate resistance to β-lactam antibiotics, e.g., T338-A for Pbp2x (SP49, SP61, and SP64) or V586-I for Pbp2A (SP61 and SP64) (12). Moreover, the dihydrofolate reductase (DHFR, *folA*) genes carry multiple mutations, causing, inter alia, an I100-L substitution in FolA, which is commonly associated with insensitivity to trimethoprim (13). Resistance to tetracycline and macrolides in *S. pneumoniae* TCH8431/19A is mediated by the Tn916-like transposon Tn2010 (AB426620.1), which contains the macrolide efflux genetic assembly (mega) element, harboring the resistance genes *tet*(M), *erm*(B), and *mef*(E)-*msr*(D) (14). Tn2010 was identified in SP61 and SP64 but not in SP49. The presence of the *mef*(E)-*msr*(D) macrolide efflux transport system may present an unspecific resistance mechanism to carolacton in SP61 and SP64.

The full-genome sequences of the three *S. pneumoniae* isolates presented here will help to understand their different susceptibilities to carolacton and evaluate possible cross-resistances in the future.

### Accession number(s).

The full-genome sequences of isolates SP49, SP61, and SP64 have been deposited in GenBank under the accession numbers CP018136, CP018137, and CP018138, respectively.
